# Promising Effects of Novel Supplement Formulas in Preventing Skin Aging in 3D Human Keratinocytes

**DOI:** 10.3390/nu16162770

**Published:** 2024-08-20

**Authors:** Angela Punzo, Matteo Perillo, Alessia Silla, Marco Malaguti, Silvana Hrelia, Diogo Barardo, Cristiana Caliceti, Antonello Lorenzini

**Affiliations:** 1 Department of Biomedical and Neuromotor Sciences, Alma Mater Studiorum, University of Bologna, 40126 Bologna, Italy; angela.punzo2@unibo.it (A.P.); matteo.perillo2@unibo.it (M.P.); antonello.lorenzini@unibo.it (A.L.); 2 Biostructures and Biosystems National Institute (INBB), 00136 Rome, Italy; 3 Department for Life Quality Studies, Alma Mater Studiorum, University of Bologna, 47921 Rimini, Italy; alessia.silla2@unibo.it (A.S.); marco.malaguti@unibo.it (M.M.); silvana.hrelia@unibo.it (S.H.); 4 NOVOS Labs, New York, NY 10017, USA; diogo@novoslabs.com

**Keywords:** aging, food supplements, 53BP1, γ-H2AX, DNA damage foci, human 3D keratinocytes, antioxidant activity

## Abstract

Dietary intervention is considered a safe preventive strategy to slow down aging. This study aimed to evaluate the protective effects of a commercially available supplement and six simpler formulations against DNA damage in 3D human keratinocytes. The ingredients used are well known and were combined into various formulations to test their potential anti-aging properties. Firstly, we determined the formulations’ safe concentration by evaluating cytotoxicity and cell viability through spectrophotometric assays. We then examined the presence of tumor p53 binding protein 1 and phosphorylated histone H2AX foci, which are markers of genotoxicity. The foci count revealed that a 24-h treatment with the supplement did not induce DNA damage, and significantly reduced DNA damage in cells exposed to neocarzinostatin for 2 h. Three of the simpler formulations showed similar results. Moreover, the antioxidant activity was tested using a recently developed whole cell-based chemiluminescent bioassay; results showed that a 24-h treatment with the supplement and three simpler formulations significantly reduced intracellular H_2_O_2_ after pro-oxidant injury, thus suggesting their possible antiaging effect. This study’s originality lies in the use of a 3D human keratinocyte cell model and a combination of natural ingredients targeting DNA damage and oxidative stress, providing a robust evaluation of their anti-aging potential.

## 1. Introduction

Biogerontology as a scientific sector has matured to the point that numerous clinical studies aim to delay the aging process [1]. While some studies have focused on single compounds or single approaches, some clinical studies have tested combinations of them [1]. The interaction of multiple compounds and/or approaches in the right balance could have a beneficial multiplicative effect and simultaneously reduce the unwanted effects that are observed with a single compound or approach at high concentrations or intensities. A recent approach in biogerontology is therefore to test mixes of compounds to find their ideal combination.

The NOVOS company has recently developed a supplement consisting of 12 ingredients, which are: (1) pterostilbene, (2) glucosamine sulfate, (3) fisetin, (4) glycine, (5) lithium aspartate, (6) calcium alpha-ketoglutarate, (7) magnesium malate, (8) vitamin C (ascorbic acid), (9) L-theanine, (10) hyaluronic acid, (11) *Rhodiola rosea* root extract, (12) ginger root extract, and a boost consisting of β-nicotinamide mononucleotide. The desiderata for the ingredients’ selection were the following:

1. They can impact the “hallmarks of aging” as defined by Lopez-Otin et al. [2];
2. They impact multiple aging mechanisms at the same time [3];
3. They have been able to extend lifespan in various animal models, hinting at conserved evolutionary pathways [4,5,6];
4. They are associated with reduced risk of mortality in humans;
5. They are associated with reduced risk of different aging-related diseases [5,7];
6. They are recognized as safe by the Food and Drug Administration (FDA), European Food Safety Agency (EFSA), and other organizations.
7. They are nature-based and not novel synthesized man-made molecules [8,9];
8. They are found in the human body, but levels decrease with age [10];
9. They have a very low or low side-effects profile [11];
10. They have been used for many decades in humans without serious side effects [12].

This study’s impact is further underscored by its demonstration of significant reductions in DNA damage and oxidative stress, suggesting the potential for effective anti-aging formulations [13,14].

Human cell cultures are a privileged study point to start testing various ingredient combinations. Over the years, several efforts have been made to study epithelial plasticity by *in vitro* and *ex-vivo* cell-based models. However, these models are limited in their capacity to mimic the *in vivo* cell microenvironment regarding cell architecture, cell-to-matrix interaction, and osmosis exchange [15]. In recent years we assisted a great expansion of innovative 3D cell models that overcame 2D cell limitations. Three-dimensional cell models allow the formation of a cellular structure, like a tissue, to facilitate interactions with the extracellular matrix and, therefore, the acquisition of a phenotype like the native one [16]. Between them, the spheroid culture system remains the most cost-effective and widely used 3D cell model [17]. We exploit the 3D human keratinocyte model, considering it a novel and more accurate representation of *in vivo* skin conditions, and its comprehensive evaluation of DNA damage and oxidative stress markers [17,18]. Among the different characteristics of the aging process, the accumulation of damage to the genetic material and oxidative stress are certainly recognized as basic elements of the deterioration process [2]. For example, we observed that, by removing fibroblasts from the skin of donors of different ages and growing the cells in culture for 10 or more passages, donor age was still significantly linked to the accumulation of genetic damage [19]. The oxidative stress theory of aging, based on the hypothesis that age-associated functional losses are due to the accumulation of reactive oxygen and nitrogen species (RONS) produced by several endogenous and exogenous processes [20], has now been discounted. Indeed, oxidative stress plays a role in aging depending on the environment—the so-called “exposome”—and it is closely related to healthspan [21]. Indeed, transgenic/knockout mice in the antioxidant defense system showed no change in lifespan, but when these models were induced to develop age-related pathologies, they showed detrimental effects in progression and/or severity. So, under chronic stress, including pathological conditions, oxidative stress plays a major role in aging, and enhanced antioxidant defenses exert an “anti-aging” action [22]. For example, oxidative stress can negatively affect the ability of stem cells to maintain their stemness [23] or, worse, produce DNA damage and contribute to carcinogenesis [24].

In this context, our study aimed to assess the possible anti-aging effects of the commercially available NOVOS food supplement and six formulas containing a maximum of five ingredients to compare the possible beneficial effects of these simpler formulas with NOVOS. To address this issue, human keratinocytes were utilized as a model, being the outermost cells of the skin, and therefore frequently exposed to DNA-damaging agents like UV light, chemicals, and physical abrasion.

Our tests primarily focused on defining the optimal concentrations to avoid possible toxicity. After establishing the concentrations that human keratinocytes (HaCaT) can tolerate, we focused on the ability to prevent DNA or oxidative damage elicited by the genotoxic agent neocarzinostatin (NCS) and the pro-oxidant menadione, respectively.

Our methodology for DNA damage focused on the immune detection of foci through the staining of tumor p53 binding protein 1 (53BP1) and the phosphorylated histone H2AX (γ-H2AX), both accepted as indirect DNA double-strand break biomarkers that, in our previous study, were found to be more sensitive than the comet assay, a direct DNA damage assay. Our previous experience showed us clearly that the superior sensitivity of the foci assays allowed us to use toxin concentrations that were a tenth of those used in the comet assay [14]. We opted for these assays because we wanted to evaluate how the tolerance of cells to mild genotoxic stress was possibly modified by the supplements’ mixtures. Different from acute forms of damage, low-to-moderate genotoxic stresses are more representative of the wear and tear of aging.

Intracellular oxidative stress investigation in 3D HaCaT was performed through a chemiluminescent probe able to selectively react with intracellular hydrogen peroxide (H_2_O_2_), one of the major members of reactive oxygen species (ROS) [25], thus monitoring its production in living cells and in the presence of possible antioxidants [26].

The data show that an “orchestra” approach of carefully selected natural compounds can have promising effects on aging-process prevention.

## 2. Materials and Methods

### 2.1. Chemicals

Phosphate-buffered saline (PBS) tabs (giving a 137 mM NaCl, 2.7 mM KCl phosphate buffer solution, pH 7.4, final concentration 0.01 M), neocarzinostatin, trypsin-EDTA, oxidative stress inductor 2-methyl-1,4-naphthoquinone (menadione), the antibiotic solution 100× (10,000 U mL^−1^ penicillin and 10 mg mL^−1^ streptomycin), Tween 20, bovine serum albumin (BSA), and ethanol were purchased from Sigma-Aldrich (St Louis, MO, USA). A stock solution of menadione (100 mM) was prepared in DMSO. The chemiluminescent probe (AquaSpark™ 510 Peroxide Probe) was provided by Biosynth Carbosynth (Staad, Switzerland). Dulbecco’s Modified Eagle Medium (DMEM) high glucose and L-glutamine were purchased from Microgem (Naples, Italy), and fetal bovine serum (FBS) was purchased from Thermo Fischer Scientific (Waltham, MA, USA). The lactate dehydrogenase (LDH) and the WST8 cell-counting kit-8 for cytotoxicity and viability assays kit (2-(2-methoxy-4-nitrophenyl)-3-(4-nitrophenyl)-5-(2,4-disulfophenyl)-2H-tetrazolium, monosodium salt) were purchased from Dojindo Molecular Technologies (Rockville, MD, USA).

### 2.2. Formulations

The company NOVOS Labs (New York, NY, USA) provided us with the commercially available food supplement NOVOS Boost which contains β-nicotinamide mononucleotide, and NOVOS Core made of 12 different ingredients: (1) pterostilbene, (2) glucosamine sulfate, (3) fisetin, (4) glycine, (5) lithium aspartate, (6) calcium alpha-ketoglutarate, (7) magnesium malate, (8) vitamin C (ascorbic acid), (9) L-theanine, (10) hyaluronic acid, (11) Rhodiola rosea root extract, and (12) ginger root extract. Data were compared with the effects of six simpler formulas (F1, F2, F3, F4, F5, and F6) containing the compounds defined in Table 1. The powder of each ingredient was provided by the NOVOS Labs company and was dissolved in water, dimethyl sulfoxide (DMSO), or a mixture of DMSO/water 1:1 (*v*/*v*) according to their solubility, the amounts present in NOVOS Core, and the concentrations found in the literature showing any biological effects [27,28,29,30,31,32,33,34] (Table 2).

NOVOS stock solution was prepared by adding 2 g of NOVOS Core and 250 mg of NOVOS Boost into a mixture of 20 mL of DMSO/water 1:1 (*v*/*v*). The solution (100 mg/mL) was sonicated for 24 min with the sonicator (Soniprep 150 Ultrasonic Disintegrator). After sonication, the mixed solution was heated to 37 °C for 10 min and centrifuged for 7 min at 1600 rpm, then the pellet was discarded and the supernatant was collected for the cell treatment.

To avoid any cell damage, the compounds solubilized in DMSO or DMSO/water 1:1 (*v*/*v*) were diluted to reach a maximum concentration of DMSO at 1% in the cell medium.

### 2.3. Cell Culture

The immortalized keratinocyte cell line from adult human skin (HaCaT) was used for all the experiments. Cells were maintained in DMEM high glucose containing 10% Fetal Bovine Serum (FBS), 2.5 mM L-glutamine, penicillin, and streptomycin at 37 °C in an atmosphere of 5% CO_2_. Cells were routinely maintained in 75 cm^2^ tissue-culture-treated flasks. Experiments in 2D HaCaT were conducted the day after cell plating.

### 2.4. 3D Human Keratinocyte Cell Model

**Figure 1:**
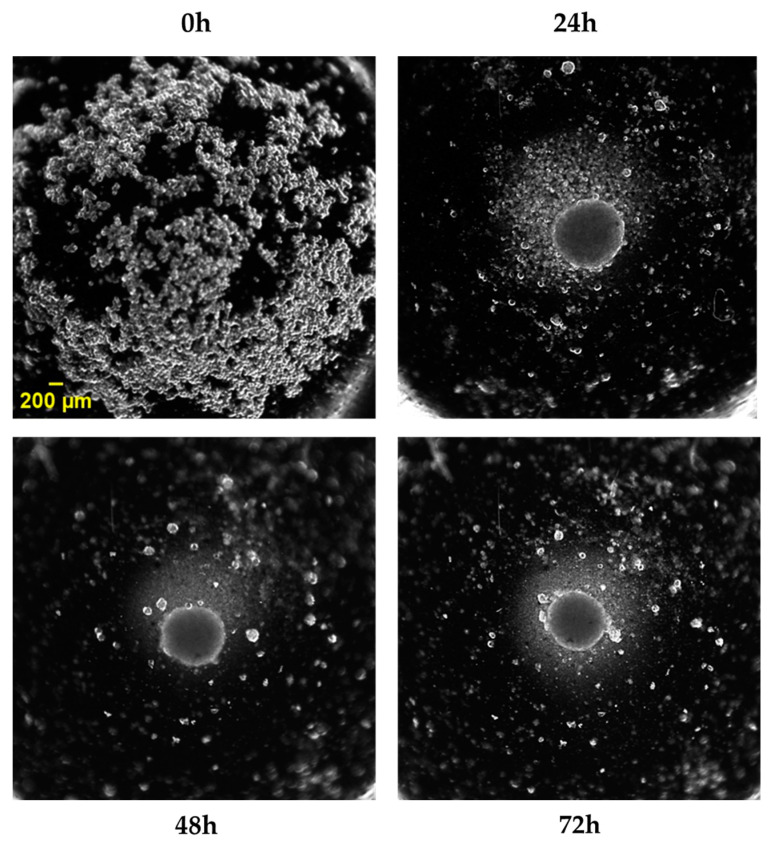
HaCaT cells (100,000 cells well^−1^) were plated in an “Ultra-Low Attachment Surface” 96-well black microtiter plate for spheroid generation, and brightfield images were acquired after 0 h, 24 h, 48 h, and 72 h using an Olympus CL40 inverted microscope connected to an electron-multiplying charge-coupled device (EMCCD) camera (ImagEM-X2, Hamamatsu, Sunayama-cho, Chuo-ku, Hamamatsu, Japan) under 4× magnification. Scale bar: 200 µm.

Cells were maintained in DMEM high glucose containing 10% FBS, 2.5 mM L-glutamine, penicillin, and streptomycin at 37 °C in an atmosphere of 5% CO_2_. To obtain 3D spheroids, HaCaT cells were seeded in the “Ultra-Low Attachment Surface” 96-well black microtiter plate (Corning, Amsterdam, The Netherlands), which consisted of a hydrophilic and neutrally charged hydrogel coating covalently bound with polystyrene surfaces (100,000 cells well^−1^). This hydrogel surface naturally inhibits non-specific interactions of the cells, eliminating unwanted cell attachment and forcing them to rest in suspension, thus allowing the 3D spheroid formation [35]. The medium culture was the same as 2D HaCaT cells and was changed daily before experiments to obtain the correct spheroid morphology [35], as shown in Figure 1. Experiments in 3D HaCaT were conducted 72 h after cell plating.

### 2.5. Cell Viability Assay

The cell viability was assessed by WST8 [2-(2-methoxy-4-nitrophenyl)-3-(4-nitrophenyl)-5-(2,4-disulfophenyl)-2H-tetrazolium, monosodium salt] (Dojindo Molecular Technologies, Kumamoto, Japan), which, in the presence of an electron mediator, is reduced by dehydrogenases in cells (as a viability biomarker) to formazan dye, which is soluble in the cell culture medium. The amount of the formazan dye generated by dehydrogenases in cells is then proportional to the number of living cells. The decrease in absorbance between the treatment after 24 h and the control was monitored at 37 °C at 450 nm using an Allsheng AMR-100 Microplate Reader (Hangzhou, China) [36]. Different sets of experiments were performed to determine dilutions of the compounds that did not affect cell viability. Briefly, HaCaT were seeded in a 96-well microtiter plate (100,000 cells well^−1^) and treated for 24 h with varying dilutions of NOVOS solution (stock 100 mg/mL in DMSO/water 1:1 (*v*/*v*)), or with individual compounds according to their solubility, as reported in Table 2. Next, we mixed the single compound solutions to obtain the six simpler formulas (as described in Table 1). Human HaCaT cells were treated with the six simpler formulas (F1, F2, F3, F4, F5, and F6) in the dilution range (1:2–1:10) for 24 h. The experiment was performed both in 2D and 3D cell models.

Differences between the means were determined by a one-way ANOVA followed by a Bonferroni correction for multiple comparisons using the GraphPad Prism Software, version 6.0 (GraphPad Software, Inc., La Jolla, CA, USA).

### 2.6. Cell Cytotoxicity: Lactate Dehydrogenase (LDH) Release

The cytotoxicity assay of LDH is based on a coupled enzymatic reaction in which LDH catalyzes the conversion of lactate to pyruvate via NAD^+^ reduction to NADH. Diaphorase reduces tetrazolium salt oxidizing NADH in the process to a red formazan product that can be measured at 490 nm. HaCaT cells were treated with different solutions of interest for 24 h in a 96-well microtiter plate (100,000 cells well^−1^). The medium was collected and the increase in absorbance between the treatment and the control was monitored at 37 °C using an Allsheng AMR-100 Microplate Reader [35]. Treatments described above were performed on HaCaT cells to find dilutions that did not have cytotoxic effects on the cells. The experiment was performed both in 2D and 3D cell models.

Differences between the means were determined by a one-way ANOVA followed by a Bonferroni correction for multiple comparisons using the GraphPad Prism Software, version 6.0 (GraphPad Software, Inc., La Jolla, CA, USA).

### 2.7. Immunofluorescence Determination of 53BP1 and γ-H2AX Foci

HaCaT (1,000,000 cells well^−1^) were seeded onto glass coverslips, pre-treated at 1:10 dilution with the six simpler formulas (F1, F2, F3, F4, F5, and F6) and NOVOS (1:250) for 24 h, then injured with NCS (0.13 μM) for 2 h. After that, the cell medium was replaced for 24 h to allow cell recovery. Cells were fixed in 70% cold ethanol for 10 min, washed once in PBS, and blocked for 30 min in 4% BSA in PBS containing Tween 20 (PBST), after which they were incubated with the primary antibody 53BP1 (Novus Biologicals, Littleton, CO, USA) in 1% BSA-PBST buffer for 1 h at room temperature in a humidified chamber. Anti-53BP1 antibody binds, in tested species, epitopes with homologies that range between 90% and 92%, compared to the human epitope [14]. Slides were washed three times in PBST and incubated with AlexaFlour555-conjugated goat antirabbit secondary antibody (Cell Signaling, Danvers, MA, USA) in 1% BSA–PBST for 1 h. Cells were washed three times, stained with Bisbenzimide (Hoechst 33342—Thermo Fisher Scientific, Waltham, MA, USA), and mounted with Vectashield mounting medium (Vector Laboratories, Burlingame, CA, USA) before analysis. Images were captured using an Olympus CL40 inverted microscope connected to an electron-multiplying charge-coupled device (EMCCD) camera (ImagEM-X2, Hamamatsu). Identifiable 53BP1 foci inside the nucleus were counted as positive foci. Nuclei were scored as containing: 53BP1 foci 0 ≤ foci < 5, 5 ≤ foci < 10, 10 ≤ foci < 20, and foci ≥ 20 [14].

This immunofluorescence protocol was also followed on 3D HacaT cells using the anti-γ-H2AX antibody. HaCaT cells were seeded, as described above, into the “Ultra-Low Attachment Surface” 96-well black microtiter plate (Corning, Amsterdam, the Netherlands) for 72 h, then pre-treated with the three simpler formulas (F1, F2, and F4) at 1:10 dilution and NOVOS solution (1:250) for 24 h, and finally injured with NCS (0.13 μM) for 2 h. After that, the cell medium was replaced with a fresh one for 24 h to allow cell recovery. Cells were fixed in 70% cold ethanol for 10 min, washed once in PBS, and blocked for 30 min in 4% BSA in PBST, after which they were incubated with the anti-γ-H2AX antibody (Anti-phospho-Histone H2A.X (Ser139) Antibody, clone JBW301, FITC conjugate, Sigma-Aldrich, Darmstadt, Germany) in 1% BSA–PBST buffer for 1 h at room temperature in a humidified chamber. Anti-γ-H2AX antibody, used for humans and mice, binds an epitope with a homology of 90% between these two species [14]. Since the antibody was already conjugated with FITC, we washed the cells three times with PBST. The fluorescence intensity (in arbitrary units) was measured using the Tecan Infinite M Nano+ (Tecan Trading AG, Männedorf, Switzerland) plate reader. Results are expressed as mean ± SD of at least three independent experiments. Differences between the means were determined by one-way ANOVA followed by the Bonferroni correction for multiple comparisons using the GraphPad Prism Software, version 6.0 (GraphPad Software, Inc., La Jolla, CA, USA).

### 2.8. Statistical Analysis and Assessment of the Formulations

The distribution of 53BP1 foci among the cells that underwent each specific combination of damage (NCS yes/no) and treatment (no treatment, NOVOS, F1, F2, F3, F4, F5, F6) was defined as the proportion of cells with: (1) fewer than 5 foci; (2) 5 to 9 foci; (3) 10 to 19 foci; (4) 20 or more foci. The pairwise differences in this distribution between cells undergoing different combinations of damage and treatments were assessed using the Chi-square test for homogeneity. Such a test allows us to evaluate whether the distribution of two samples across various levels (in this case, the distribution of cells across different levels of foci numerosity) is equal or not. In total, 29 pairwise comparisons were performed with the following settings:

- Non-damaged and non-treated cells (Ctrl) vs. non-damaged and treated cells (NOVOS or F#);
- Non-damaged and non-treated cells (Ctrl) vs. damaged cells, both non-treated (Ctrl_NCS) and treated (NOVOS_NCS or F#_NCS);
- Non-damaged treated cells (NOVOS or F#) vs. damaged treated cells (NOVOS_NCS or F#_NCS);
- Damaged non-treated cells (Ctrl_NCS) vs. damaged treated cells (NOVOS_NCS or F#_NCS).

F# stands for any of the 6 formulations.

The *p*-values resulting from the tests indicated the level of statistical significance of the difference between the observed distributions and the distributions expected in the case of homogeneity (i.e., in the case of no difference in distribution). A corrected significance level $\alpha_{c}$ = 0.002 was set, resulting from the application of the Bonferroni correction to a baseline significance level α = 0.05.

Since experiments in 3D cultures are labor intensive, we opted to reduce the number of formulations to be tested further, excluding at least a couple of them. To do so, we performed some preliminary analyses and ranked the 6 proposed compound formulations based on their effectiveness and complexity.

The effectiveness was assessed by quantifying the impact of the formulation on the distribution of 53BP1 foci among cells that underwent damage. We started from a dataset reporting the observed foci distribution for each combination of damage and treatment. That dataset contained 4 variables, each counting the proportion of cells presenting a specific level of foci (levels reported above), and 16 observations, each corresponding to one of the combinations of damage and treatment (reported above).

A principal component analysis (PCA) was performed to reduce the dimensionality of the dataset, and the feature space described by the resulting first two principal components was considered to assess the formulations.

In that space, the two clusters corresponding to damaged and non-damaged cells were defined, as were their centroids. The weighted Euclidean distance between each of the data points of the “damaged cells” cluster with the centroid of the “non-damaged cells” cluster was computed using the following formula:

$$d_{p,c}=\sqrt{w_{1}{\left(p_{1}-c_{1}\right)}^{2}+w_{2}{\left(p_{2}-c_{2}\right)}^{2}}$$

The weight $w_{i}$ referred to the *i*-th principal component was computed as follows:

$$w_{i}=\frac{{ve}_{i}}{{ve}_{1}+{ve}_{2}}$$

$p_{i}$ and $c_{i}$ represent the value for the *i*-th principal component of the generic point p and of the centroid c, respectively. ${ve}_{i}$ is the proportion of sample variance explained by the *i*-th principal component.

The resulting distance was used as a measure of the effectiveness of the compound formulations. A lower distance suggests that a formulation is more effective since it means that the foci distribution for cells damaged and then treated is more similar to the distribution of non-damaged cells.

The complexity of the formulations was assessed by counting the number of ingredients used for each formulation, to prioritize the ones with fewer ingredients.

### 2.9. Quantification of Intracellular H_2_O_2_ in 2D and 3D HaCaT Cells

The intracellular H_2_O_2_ production was estimated in 2D and 3D HaCaT cells by using a chemiluminescent (CL) cell-based bioassay previously developed in our lab [26,35,37]. Briefly, HaCaT cells (100,000 cells well^−1^) were plated in a 96-well black microtiter plate with a clear bottom or into the “Ultra-Low Attachment Surface” 96-well black microtiter plate depending on the 2D, or 3D model, respectively. Next, cells were treated with serial dilutions of the formulations F1, F2, F4 (range 1:10–1:1000), and NOVOS solution (range 1:250–1:156,250) for 24 h. Then, the cell medium was removed and 100 µL of the H_2_O_2_-CL probe working solution (10 µM of CL probe in PBS, pH 7.5, final concentration 5 µM) was added. After the incubation for 20 min at 37 °C, 100 µL of the oxidant agent menadione (final concentration 25 µM in PBS, pH 7.4) was dispensed into each well to induce intracellular H_2_O_2_ production. The CL emission signal was monitored for 60 min using a Varioskan Flash (Thermo Scientific, Waltham, MA, USA) luminometric plate reader. The temperature was maintained at 37 °C during the measurement.

## 3. Results and Discussion

### 3.1. Safety of NOVOS and the Six Simpler Formulas in Human Keratinocytes

Cell-based assays are appealing tools for evaluating biological activities in cosmetics since they reduce animal testing.

**Figure 2:**
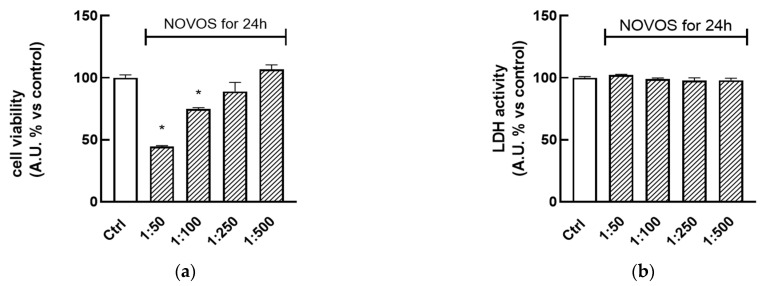
HaCaT (100,000 cells well^−1^) were plated in a 96-well microtiter plate and treated for 24 h with NOVOS solution (100 mg/mL in DMSO/water 1:1 (*v*/*v*)) (dilution range 1:50–1:500). Cell viability (**a**) and cytotoxicity (**b**) were assessed using WST8 and LDH assays, respectively. Untreated HaCaT cells served as a control (Ctrl). Results are expressed as means ± SD of three independent experiments performed in triplicate. * *p* < 0.001 significantly different from the control.

To determine the safety of NOVOS and the six simpler formulas coded F1, F2, F3, F4, F5, and F6, cell viability and cytotoxicity were evaluated through the conventional bioassays based on the water-soluble tetrazolium salt WST-8 and lactate dehydrogenase (LDH) release, respectively. HaCaT cells were treated with NOVOS solution (dilution range 1:50–1:500) for 24 h, showing that dilutions 1:250 and 1:500 did not significantly lower cell viability and proliferation, and were utilized for further analysis (Figure 2a). On the other hand, dilutions at 1:50 and 1:100 significantly reduced cell viability so they were not used for further experiments. The same treatments were performed in parallel to find the dilutions that did not exert cytotoxic effects. Results indicated that 24-h treatments (range 1:50–1:500) were not cytotoxic, as indicated by the absence of increasing LDH levels in the cell medium (Figure 2b). According to these results, NOVOS dilution at 1:250 was used for subsequent experiments since it was demonstrated to be the most concentrated and safest one.

Using the same approach, these bioassays were performed for each of the compounds (calcium alpha-ketoglutarate, di-magnesium malate, pterostilbene, lithium aspartate, glycine, glucosamine sulfate, fisetin, spermidine, and trehalose) present in the six simpler formulas to determine the concentration that did not affect cell viability and did not exhibit cytotoxic effects.

**Figure 3:**
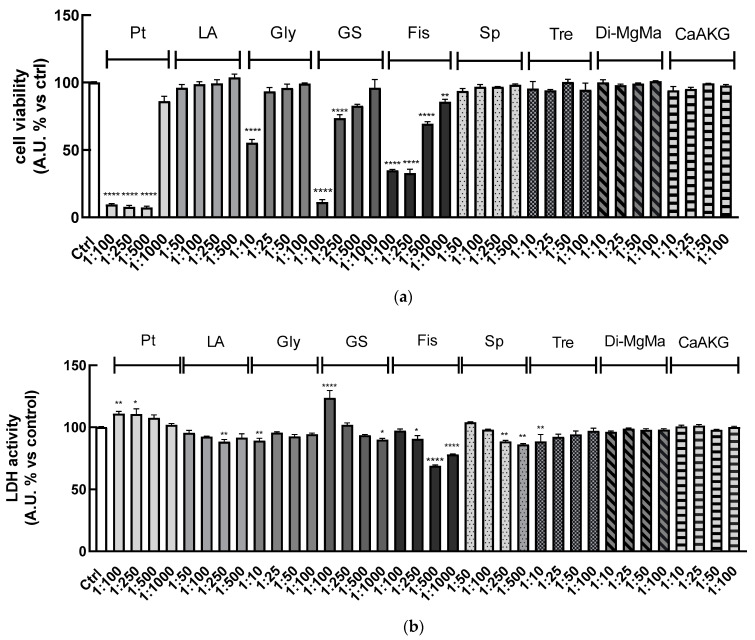
HaCaT (100,000 cells well^−1^) were plated in a 96-well microtiter plate and treated for 24 h with the single ingredients of the six simpler formulas (calcium alpha-ketoglutarate (CaAKG), di-magnesium malate (Di-MgMa), pterostilbene (Pt), lithium aspartate (LA), glycine (Gly), glucosamine sulfate (GS), fisetin (Fis), spermidine (Sp), and trehalose (Tre) at different dilutions according to their solvent (water: 1:10; 1:25; 1:50 and 1:100; DMSO: 1:100; 1:250; 1:500 and 1:1000; DMSO/water 1:1 (*v*/*v*) 1:50, 1:100; 1:250 and 1:500). Cell viability (**a**) and cytotoxicity (**b**) were assessed using WST8 and LDH assays, respectively. Untreated HaCaT cells served as a control (Ctrl). Results are expressed as means ± SD of three independent experiments, each performed in triplicate. * *p* < 0.05, ** *p* < 0.001, **** *p* < 0.0001 significantly different from the control.

Results showed that lithium aspartate, spermidine, trehalose, di-magnesium malate, and calcium alpha-ketoglutarate did not reduce cell viability for all the dilutions tested. Pterostilbene at dilution 1:1000 did not reduce cell viability, while increased concentrations greatly affected cell viability. Only at dilution 1:10 did glycine reduce cell viability, while the other ones were safe. The more concentrated 1:100 and 1:250 dilutions of glucosamine sulfate reduced cell viability, while 1:500 and 1:1000 did not. Fisetin at all dilutions tested seemed to reduce cell viability, but these results can be partially related to the yellow-colored solutions of the compound, which can interfere with the absorbance reading (Figure 3a).

On the other hand, the LDH assay indicated that all the treatments performed (except for 1:100 glucosamine sulfate and 1:100, 1:250 pterostilbene) were not cytotoxic after 24 h, as shown in Figure 3b.

Based on these results, we tested the concentrations reported in the second column of Table 3 for further cell experiments.

**Figure 4:**
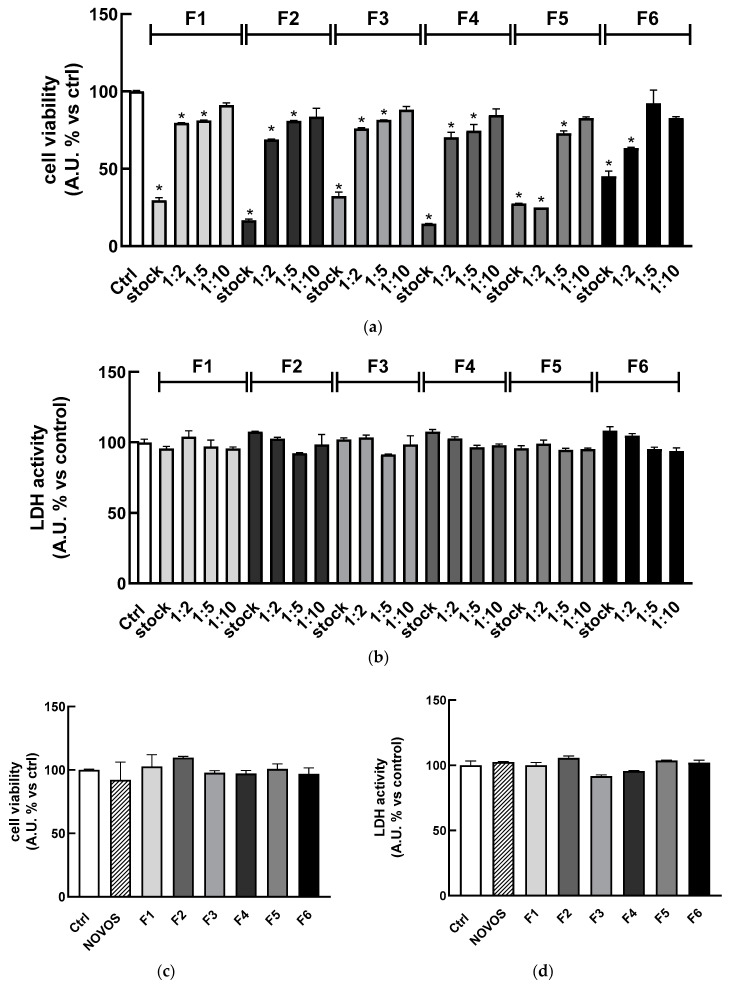
HaCaT (100,000 cells well^−1^) were treated for 24 h with the six simpler formulas (F1, F2, F3, F4, F5, and F6) at different dilutions (stock, 1:2, 1:5, and 1:10). Cell viability (**a**) and cytotoxicity (**b**) were assessed using WST8 and LDH assays, respectively. HaCaT spheroids were treated for 24 h with the six simpler formulas (1:10). Next (**c**) cell viability and (**d**) cytotoxicity assays were performed. Untreated HaCaT served as a control (Ctrl). Results are expressed as means ± SD of three independent experiments performed in triplicate. * *p* < 0.001 significantly different from the control.

HaCaT cells were treated for 24 h with the combinations reported in Table 1 (F1, F2, F3, F4, F5, and F6), and then cell viability and cytotoxicity were determined as previously described. The results shown in Figure 4a indicated that the combination of compounds in their “safe concentration” significantly reduced the cell viability compared to the control (stock). These results were not surprising since the cumulative concentration of compounds in the simpler formulas is higher with respect to the isolated ones and the use of a multicomponent formula is expected to lead to complex interactions between compounds and multiple targets, leading to a different efficacy [38].

So, to avoid any detrimental effect, HaCaT were treated with different dilutions (range 1:2–1:10) of the six simpler formulas (F1, F2, F3, F4, F5, and F6) for 24 h, observing that the most diluted (1:10) solutions did not reduce cell viability as well as 1:5 dilution did, just for the F6 formula (Figure 4a). Regarding the cytotoxicity, Figure 4b shows that each formula (range 1:1–1:10) was not cytotoxic after 24 h of treatment, as highlighted by the lack of significant increase in LDH levels in the cell medium. These results were confirmed by using the Trypan Blue Exclusion Test (Table S1) to detect live and dead cells treated with the six simpler formulas (1:10 dilution) and NOVOS (1:250 dilution) before and after NCS injury.

Two-dimensional skin cell cultures have been used for decades as one of the most appealing tools for evaluating a plethora of biological activities in the cosmetic area since they provide human-relevant predictive information. Indeed, skin cell culture allows us to avoid or reduce *in vivo* experiments in compliance with the guiding principle of the “Three Rs”: Replacement, Reduction, and Refinement. However, 2D cell cultures harbor several limitations since they do not reflect the natural structure of tissues and, as such, cell–cell, and cell–environment interactions are not fully represented. This has driven the development of 3D cell culture systems that overcome the intrinsic limitations of conventional 2D cells, more closely mimicking the complex heterogeneity of cell morphology.

Three-dimensional HaCaT were grown as previously described in the Materials and Methods Section, according to the cell culture protocol reported by Klicks et al. [39] and already validated and routinely used in our lab [35]. Once the spheres were formed, 3D HaCaT spheroids were treated for 24 h with the six simpler formulas (1:10 dilution) and NOVOS (1:250 dilution). Results showed that treatments did not reduce cell viability and were not cytotoxic (Figure 4c,d), thus the dilution 1:10 of the simpler formulas and 1:250 of NOVOS were used for further experiments (final concentrations are reported in the third column of Table 3).

### 3.2. DNA Damage Evaluation Using 53BP1 Foci Formation in Human Keratinocytes

53BP1 foci are a recognized marker for DNA damage. In a large investigation of skin-derived fibroblasts from 100 human donors aged 18 to over 90 years old, we reported a positive correlation between donor age and 53BP1 foci abundance, and also a positive association between the presence of 53BP1 foci and the number of micronuclei [19]. Micronuclei are indeed dosed to detect genotoxic damage in established protocols drawn up by the Organisation for Economic Co-operation and Development (OECD).

Two-dimensional HaCaT were pre-treated with the six simpler formulas F1, F5, F3, F2, F4, and F6 (1:10 dilution) and NOVOS (1:250 dilution) for 24 h, and then injured with NCS (0.13 μM) for 2 h.

Especially in keratinocytes, given the exposure of skin cells to environmental stressors like UV radiation, the proper function of DDR proteins is essential for preventing genomic instability. 53BP1 plays a key role: deficiencies of the transcript or mutations in the gene can lead to increased sensitivity to DNA damage and defective DNA repair mechanisms, thus potentially contributing to skin disorders and cancers [40].

**Figure 5:**
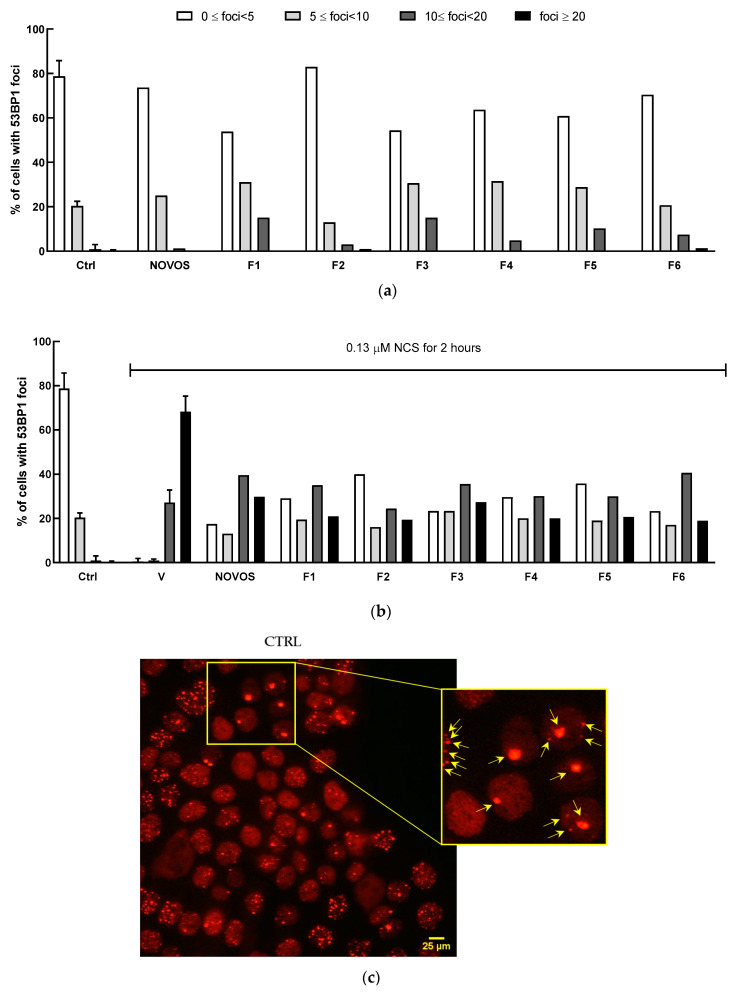

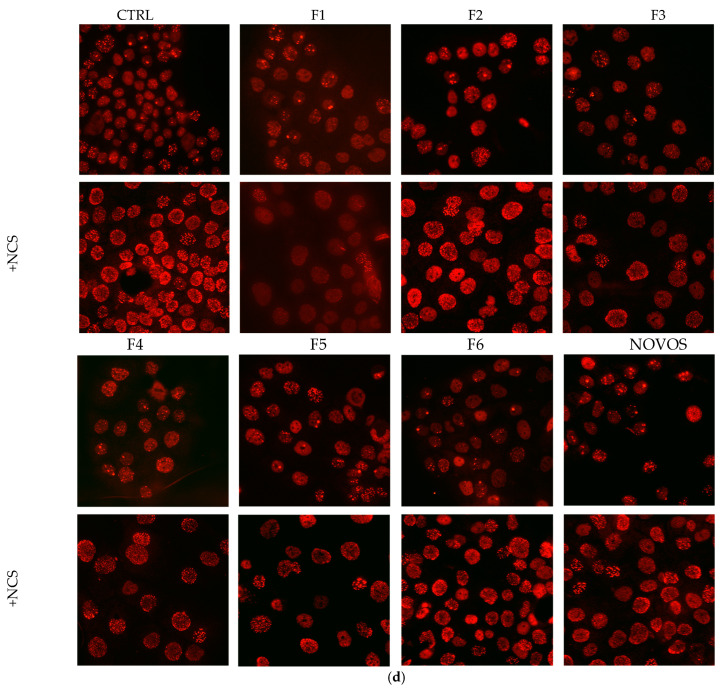
HaCaT (100,000 cells well^−1^) were pre-treated for 24 h with NOVOS (1:250 dilution) and the six simpler formulas F1, F2, F3, F4, F5, and F6 (1:10 dilution) (**a**), then injured with 0.13 μM NCS for 2 h (**b**) and fixed after 24 h as previously described. Untreated HaCaT cells were included as controls. V, positive control cells treated with 0.13 μM NCS for 2 h. Cells scored for 53BP1 foci were grouped into 0 ≤ foci < 5, 5 ≤ foci < 10, 10 ≤ foci < 20, and foci ≥ 20 foci per nucleus. The histogram bars show the percentage of nuclei with 53BP1 foci for every group. Nuclear arrangement of the 53BP1 protein in spontaneously occurring DNA lesions; 53BP1 foci are marked with yellow arrows (**c**). Representative images of cells immunostained with anti-53BP1 antibody (**d**). Scale bar: 25 µm.

DNA damage analysis, as 53BP1 positive foci counting, revealed that NOVOS treatment did not induce foci formation, while all six simpler formulas had light detrimental effects. NOVOS treatment significantly prevented the damage burden in the heavily damaged NCS-treated cells. At the same time, all the simpler formulas significantly prevented DNA damage, observed as reduced foci numbers, even with a stronger effect compared to the NOVOS’ original formulation itself (Table 4 and Figure 5a, b).

### 3.3. Assessment and Selection of the Formulations

All the Chi-square tests for homogeneity showed statistically significant differences (*p* < 0.002) in the distribution of the number of foci between cells undergoing different combinations of damage and treatment, except for the comparison between Ctrl and NOVOS, which did not show a statistically significant difference (*p* = 0.23). The exact Chi statistics and *p*-values are reported in Table 4 and Table S2.

The PCA conducted on the dataset reporting the foci distribution of the 16 combinations of damage and treatment yielded the following results: the first two components accounted for 94.2% of the sample variability (79.9% for PC1 and 16.2% for PC2). As shown in Figure S1, PC1 separates the combinations yielding a high number of foci (x ≥ 10) from the ones yielding a low number of foci (x < 10), while PC2 separates the combinations yielding an extremely high or low number of foci (≤5 or >20) from the ones yielding an intermediate number of foci (5 ≤ x < 20).

**Figure 6:**
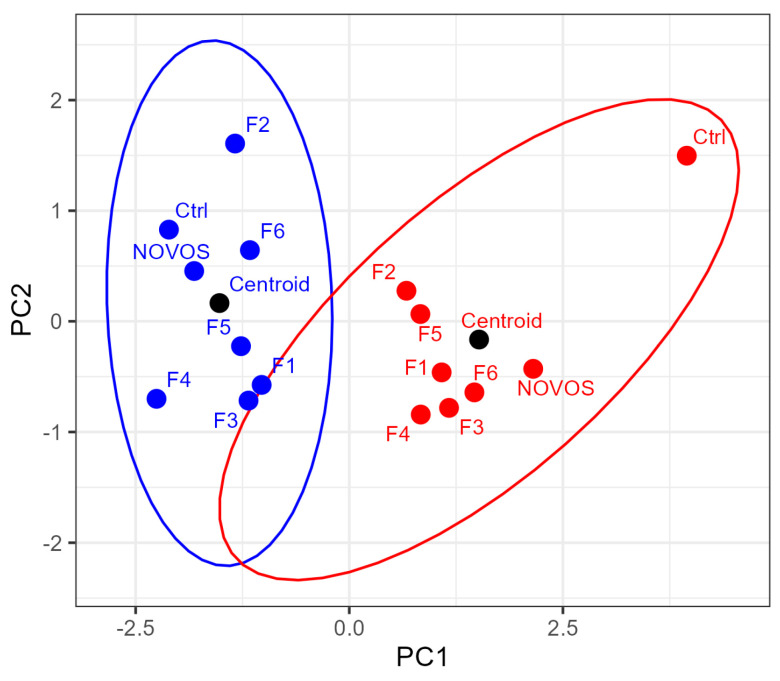
Score plot showing the position of the formulations and the centroids in the two-dimensional space defined by the two principal components PC1 and PC2. The blue points refer to cells that did not undergo NCS damage, while the red points refer to cells that underwent NCS damage. The ellipses define the 95% confidence area of the average values of PC1 and PC2 for the points belonging to each cluster (with and without NCS damage).

The score plot in Figure 6 shows the position of the formulations and of the centroids in the two-dimensional space defined by the two principal components PC1 and PC2. The resulting effectiveness ranking of the formulations based on the weighted Euclidean distance from the centroid of the non-NCS group is: F2, F5, F4, F1, F3, and F6.

With regard to complexity, F1 and F4 were the formulations with the fewest ingredients (three), followed by F2 and F4 (four ingredients), and F5 and F6 (five ingredients).

Based on this, we decided to select the following formulations for further testing: F2, F4, and F1. Despite showing slightly poorer performance, F1 was selected over F5 because of the lower number of ingredients. The weighted Euclidean distances and the number of ingredients for each formulation are reported in Table S3.

### 3.4. Antioxidant Activity of the Formulations in Human 2D and 3D Keratinocytes

**Figure 7:**
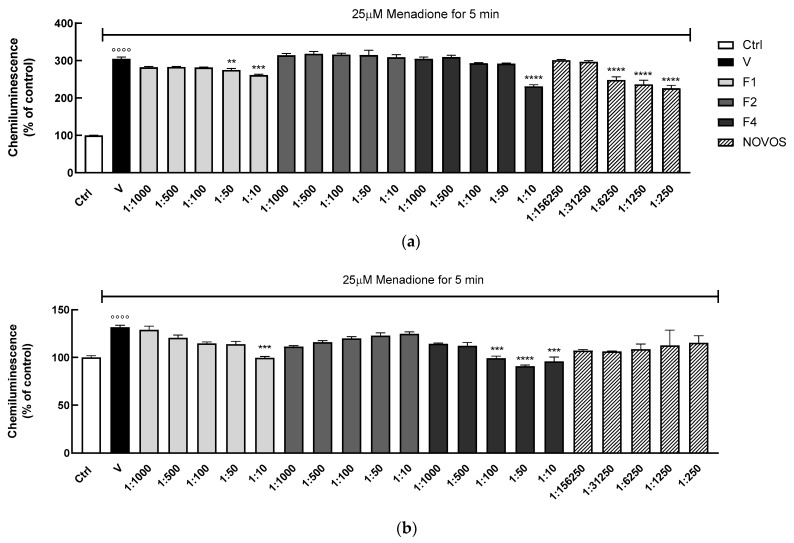
HaCaT (100,000 cells well^−1^) were pre-treated for 24 h with NOVOS (dilution range 1:250–1:156,250) and the three simpler formulas, F1, F2, and F4 (dilution range 1:10–1:1000). The chemiluminescence signal was analyzed after 5 min of 25 μM menadione injuring in human 2D (**a**) and 3D HaCaT (**b**) cells. Results are expressed as mean ± SD of three independent experiments. °°°° *p* < 0.0001 significantly different from the ctrl. ** *p* < 0.01, *** *p* < 0.0005, and **** *p* < 0.0001 significantly different from V. Ctrl (control, untreated cells); V (control cells in the presence of menadione).

The antioxidant activity of the most promising formulations was assessed previously in 2D HaCaT, and then in the 3D cell model, to closely mimic the human skin. The detection of intracellular ROS in living cells represents a challenge since ROS are short-lived and their direct detection is seldom feasible. Intracellular H_2_O_2_ levels can reasonably reflect a general change in intracellular ROS production because various ROS are converted to H_2_O_2_ within cells, therefore the selective measurement of H_2_O_2_ over different ROS is critical for the accurate evaluation of oxidative stress. In our study, the intracellular production of H_2_O_2_ in human living cells was analyzed by the CL bioassay previously developed in our laboratory [26], which overcame several limitations of other bioassays. Two-dimensional and three-dimensional HaCaT were treated with increasing concentrations of F1, F2, and F4 formulas (range 1:10–1:1000) and NOVOS (range 1:250–1:156,250) for 24 h, and then injured with the pro-oxidant menadione [41]. Pre-treatments of 2D HaCaT over 24 h showed that only the formulations F1 (1:50 and 1:10), F4 (1:10), and NOVOS (1:16,250, 1:1250 and 1:250) significantly reduced intracellular H_2_O_2_ production after 5 min of menadione injuring (Figure 7a).

These data were confirmed in the 3D model, showing that only F1 (1:10) and F4 (1:100, 1:50, and 1:10) significantly reduced intracellular H_2_O_2_ production (Figure 7b). The correlation between oxidative stress and a decrease in healthspan is very well reported, at least in part due to a decline in mitochondrial function that causes elevated ROS levels and an impairment in antioxidant defense [13]. Our results indicate that F1 and F4 had a significant effect in reducing intracellular ROS production in 2D and 3D human keratinocytes.

### 3.5. Evaluation of γ-H2AX Foci Formation in Human 3D Keratinocytes

Double strand breaks (DSBs) are the most harmful DNA lesions a cell can encounter; nuclear foci of γ-H2AX or 53BP1, individually or in combination, are widely used as DNA DSBs markers and their abundance has been reported to correlate very closely with the degree of genotoxic insult, although some precautions must be kept in mind when comparing data obtained on different species [14].

**Figure 8:**
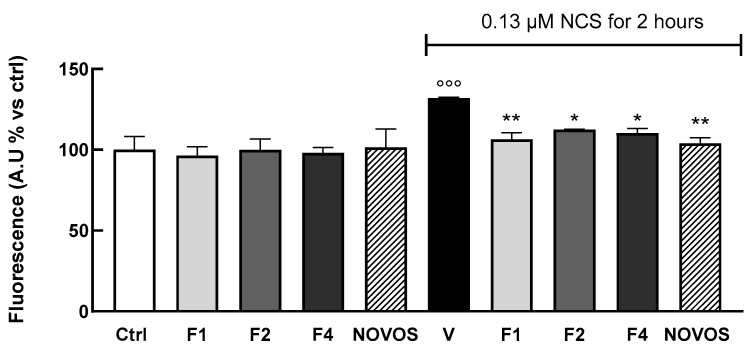
Three-dimensional HaCaT (100,000 cells well^−1^) were pre-treated with NOVOS (1:250 dilution) and the simpler formulas F1, F2, and F4 (1:10 dilution) for 24 h, then were injured with 0.13 μM NCS for 2 h and fixed. Untreated cells were included as controls. Results are expressed as mean ± SD of three independent experiments. °°° *p* < 0.0001 significantly different from the ctrl. * *p* < 0.05 and ** *p* < 0.005 significantly different from NCS. Ctrl (control, untreated cells); V (control cells in the presence of 0.13 μM NCS for 2 h).

To test if the simpler formulas and NOVOS pre-treatment significantly reduced DNA damage, γ-H2AX foci formation was also evaluated in 3D HaCaTs. Results showed that a 24-h treatment of F1, F2, and F4 (1:10 dilution), and NOVOS (1:250 dilution), significantly reduced DNA damage compared with cells injured with 0.13µM NCS for 2 h (Figure 8). These data confirm the promising effect of NOVOS, F1, F2, and F4 in counteracting, at least in part, skin aging. Of interest, NOVOS seems to have beneficial effects at a cumulative concentration significantly lower than the simpler formulas, underling possible synergistic effects between the compounds.

γ-H2AX is a pivotal marker and mediator of the DNA damage response. It acts as a recruitment platform for other DNA damage response (DDR) proteins, including MDC1, 53BP1, and the MRN complex (MRE11-RAD50-NBS1), amplifying the DNA damage signal and facilitating the recruitment of repair machinery to the damage site [18].

Our results confirm the ability of NOVOS and the simpler formulas to counteract NCS injury, possibly by pre-alerting fundamental DDR proteins.

## 4. Conclusions

A recent cornerstone comparative biology study has clearly demonstrated that an organism can support a maximum number of mutations during its lifetime [42]. Our species, which is equipped with a high longevity quotient [43], is indeed also equipped with a notable capacity for recognizing genetic damage, [44] and this allows it to effectively activate repair mechanisms or other strategies to minimize damage consequences at the tissue level [45]. This type of evidence suggests that appropriate cocktails of substances, such as those tested in this work, which can activate the cellular defenses against genetic and oxidative damage, could slow down the fundamental molecular mechanisms underlying the aging process and thus reduce the burden of diseases associated with it. Efficient DDR, in addition, is crucial for preventing mutations that could lead to skin cancers and other disorders.

Our results on foci formation in 3D human keratinocytes suggest that the tested new formulations may act through hormetic mechanisms. Hormesis has been proposed as a potential explanation for a variety of phytochemicals [46] that will act by provoking mild stresses that prompt cells to sustain subsequent major insults. It is interesting to note, in this regard, that the original complete NOVOS formulation has, instead, a notable DNA damage-preventing action without showing any mild DNA damaging effect.

Our study has some limitations: it lacks a direct measure of DNA damage. It also lacks analyses required to clarify the different modes of action and the major cellular mechanisms involved in the traditional and new formulations. Moving forward, it will be important to add direct determinations of DNA damage and to start probing key pathways that may be responsible for the hormetic response.

On the other hand, our study also has strengths: its impact is underscored by its demonstration of significant reductions in DNA damage and oxidative stress in 2D and 3D cultures, suggesting the potential for effective anti-aging formulations of natural ingredients.

## Figures and Tables

**Table 1:** Formulation (F) compositions.

F	Calcium Alpha-Ketoglutarate	Di-Magnesium Malate	Pterostilbene	Lithium Aspartate	Glycine	Glucosamine Sulfate	Fisetin	Spermidine	Trehalose
1			X			X	X		
2	X		X			X	X		
3			X		X	X	X		
4			X	X			X		
5		X	X		X	X	X		
6	X			X			X	X	X

**Table 2:** Solvents used and stock concentration of compounds.

Compounds	Water	DMSO	DMSO/Water 1:1 (*v*/*v*)	Stock Concentration [M]
Calcium alpha-ketoglutarate	X			80 mM
Di-magnesium malate	X			40 mM
Pterostilbene		X		390 mM
Lithium Aspartate			X	1000 mM
Glycine	X			2950 mM
Glucosamine Sulfate	X			550 mM
Fisetin		X		349 mM
Spermidine			X	688 mM
Trehalose			X	730 mM

**Table 3:** Compound concentrations that did not affect cell viability and were not cytotoxic on cells treated with the six simpler formulas.

Compound	Safe Concentration for the Individual Compound [M]	Safe Concentration of Compounds in Each Simpler Formula [M]
Calcium alpha-ketoglutarate	4 mM	0.4 mM
Di-magnesium malate	4 mM	0.4 mM
Pterostilbene	390 μM	39 μM
Lithium Aspartate	10 mM	1 mM
Glycine	118 mM	11.8 mM
Glucosamine Sulfate	2.2 mM	0.2 mM
Fisetin	698 μM	69.8 μM
Spermidine	13.76 mM	1.3 mM
Trehalose	73 mM	7.3 mM

**Table 4:** Percentage of cells with 53BP1 foci and *p*-values resulting from the Chi-square tests for homogeneity. The values reported in the column “Test 1” refer to the comparison between damaged and non-damaged cells treated with the same formulation; the column “Test 2” refers to the comparison between each combination and Ctrl; the column “Test 3” refers to the comparison between each formulation with NCS and Ctrl NCS. In order to respect the assumptions on the Chi-square test, the tests were performed by merging the last two foci levels (“10 to 19 foci” and “20 or more foci”) into one unique level (“10 foci or more”). Hence, the reference distribution for all the tests is a chi-square with 2 degrees of freedom. The stars represent the level of significance of the test. All the comparisons, except for the one between Ctrl and NOVOS show statistically significant differences with *p*-value < 0.002.

Treatment	% Cells with 0 ≤ Foci < 5	% Cells with 5 ≤ Foci < 10	% Cells with 10 ≤ Foci < 20	% Cells with Foci ≥ 20	Test 1	Test 2	Test 3
Ctrl	78.73	20.36	0.90	0.00	5.2 × 10^−177^ ***	\\\	\\\
Ctrl_NCS	0.47	0.95	27.25	68.25		5.2 × 10^−177^ ***	\\\
NOVOS	73.68	25.00	1.32	0.00	1.19 × 10^−88^ ***	0.23	\\\
NOVOS_NCS	17.48	13.11	39.56	29.85		1.7 × 10^−101^ ***	8.90 × 10^−29^ ***
F1	53.85	31.07	15.09	0.00	1.96 × 10^−17^ ***	2.73 × 10^−18^ ***	\\\
F1_NCS	29.10	19.49	35.03	20.90		3.37 × 10^−95^ ***	1.3 × 10^−170^ ***
F2	82.95	12.98	3.05	1.02	4.69 × 10^−41^ ***	3.55 × 10^−4^ **	\\\
F2_NCS	40.00	16.11	24.44	19.44		4.31 × 10^−51^ ***	9.66 × 10^−64^ ***
F3	54.37	30.60	15.03	0.00	6.57 × 10^−34^ ***	3.10 × 10^−18^ ***	\\\
F3_NCS	23.36	23.36	35.61	27.35		1.63 × 10^−81^ ***	4.84 × 10^−44^ ***
F4	63.68	31.49	4.83	0.00	2.98 × 10^−50^ ***	4.33 × 10^−7^ ***	\\\
F4_NCS	29.68	20.09	30.14	20.09		1.51 × 10^−67^ ***	7.44 × 10^−56^ ***
F5	60.86	28.86	10.29	0.00	2 × 10^−26^ ***	2.67 × 10^−11^ ***	\\\
F5_NCS	35.81	19.03	30.00	20.65		8.16 × 10^−58^ ***	3.73 × 10^−56^ ***
F6	70.44	20.72	7.46	1.38	1.38 × 10^−49^ ***	3.35 × 10^−7^ ***	\\\
F6_NCS	23.31	17.07	40.65	18.97		1.48 × 10^−80^ ***	9.44 × 10^−41^ ***

**: *p*-value < 0.0003; ***: *p*-value < 3 × 10^−5^—these two values correspond to the Bonferroni-corrected significance levels 0.01 and 0.001.

## Data Availability

The raw data supporting the conclusions of this article will be made available by the authors on request.

## Supplementary Materials

The following supporting information can be downloaded at: https://www.mdpi.com/article/10.3390/nu16162770/s1. Table S1. HaCaT were treated for 24 h with the six simpler formulas (1:10 dilution) and NOVOS (1:250 dilution), then injured with 0.13 μM NCS for 2 h. The Trypan blue exclusion test was performed three times to evaluate live and dead cells. Each time, 1000 cells were analyzed, for a total of 3000 cells per treatment. Results are expressed as dead cells (%) ± standard deviation (SD) by calculating the ratio between the total number of dead cells per ml of aliquot and the total number of cells per ml of aliquot. Eight two-proportion z-tests were performed to assess the difference in proportion of dead cells before and after NCS damage (Test 1); seven tests of the same type were carried out to assess the difference after NCS damage between the control group and each treatment (Test 2). All the tests had a very low *p*-value (9.60 × 10^−292^ or lower for Test 1, 2.29 × 10^−55^ or lower for Test 2), making all the tested differences statistically significant, even after applying the Bonferroni correction for multiple comparisons. Table S2. Results of the 28 pairwise Chi-square tests for homogeneity, assessing the similarities in distribution of foci among cells that underwent different types of damage/treatment. The reference distribution for all the tests is a chi-square with 2 degrees of freedom. All the comparisons showed statistically significant differences with *p*-value < 0.002, except for the one between Ctrl and NOVOS. Figure S1. Loading plot showing the correlation between each of the original variables describing the foci distribution and the first two components resulting from the PCA. More cells with 10 or more foci resulted in a higher value of PC1, while more cells with fewer than 5 or more than 19 foci resulted in a higher value of PC2. Table S3. Number of ingredients in each formulation and corresponding weighted Euclidean distance from the centroid of the “non-damaged cells” cluster. A lower distance suggests a higher effectiveness of the formulation.
