# Supplementary material for: Promising Effects of Novel Supplement Formulas in Preventing Skin Aging in 3D Human Keratinocytes
Source: Nutrients. 2024 Aug 20;16(16):2770. doi: 10.3390/nu16162770 (PMC11356847; doi:10.3390/nu16162770)
Supplement: Supplementary file 1 [file nutrients-16-02770-s001.zip › nutrients-3117609-supplementary.pdf]

nutrients MDPI

Supplementary materials

Tables and Figures about the statistical analysis and formulations selection

Trypan blue exclusion test

HaCaT cells were treated for 24h with the six simpler formulas F1-F6 (1:10 dilution) and NOVOS (1:250 dilution), then injured with 0.13 μM NCS for 2h. Cells were washed in PBS and detached in Trypsin EDTA. Next, 1 part of the cell suspension was mixed with the same volume of 0.4% trypan blue stain (Thermo Fisher), and 10 μL of the sample mixture was loaded into the sample slide, per chamber, and live/ dead cells were acquired using Countess™ II Automated Cell Counter (Thermo Fisher).

Table S1. HaCaT were treated for 24h with the six simpler formulas (1:10 dilution) and NOVOS (1:250 dilution), then injured with 0.13 μM NCS for 2h. Trypan blue exclusion test was performed three times, to evaluate live and dead cells. Each time, 1000 cells were analyzed, for a total of 3000 cells per treatment. Results are expressed as dead cells (%) ± standard deviation (SD), by calculating the ratio between the total number of dead cells per ml of aliquot and the total number of cells per ml of aliquot. 8 two-proportions z-tests were performed to assess the difference in proportion of dead cells before and after NCS damage (Test 1); 7 tests of the same type were carried out to assess the difference after NCS damage between the control group and each treatment (Test 2). All the tests had a very low p-value (9.60E-292 or lower for Test 1, 2.29E-55 or lower for Test 2), making all the tested differences statistically significant also after applying the Bonferroni correction for multiple comparisons.

Group	Dead cells before NCS (% value ± SD)	Dead cells after NCS (% value ± SD)	Test 1 Before vs after NCS p-value and significance level	Test 2 After NCS vs Ctrl p-value and significance level
Ctrl	6 ± 2	82 ± 10	<3.4E-308***	\ \ \
NOVOS	8 ± 3	64 ± 13	<3.4E-308***	2.29E-55***
F1	7 ± 2	56 ± 6	<3.4E-308***	7.70E-105***
F2	9 ± 4	59 ± 9	<3.4E-308***	1.00E-84***
F3	12 ± 2	63 ± 12	<3.4E-308***	8.20E-61***
F4	10 ± 3	54 ± 8	9.6E-292***	2.90E-119***
F5	7 ± 3	51 ± 12	3.4E-308***	1.90E-142***
F6	9 ± 3	61 ± 6	<3.4E-308***	2.39E-72***

*: p-value < 0.003; **: p-value < 0.0007; ***: p-value < 7E-5 - These three values correspond to the Bonferroni-corrected significance levels 0.05, 0.01, 0.001 (for 15 comparisons).

Table S2. Results of the 29 pairwise Chi-square tests for homogeneity, assessing the similarities in the distribution of foci among cells that underwent different types of damage/treatment. The reference distribution for all the tests is a chi-square with 2 degrees of freedom. All the comparisons show statistically significant differences with p-value < 0.002, except for the one between Ctrl and NOVOS.

Group 1	Group 2	Chi-square value	p-value	Significance level
Ctrl	Ctrl_NCS	811.82	5.20E-177	***
Ctrl	NOVOS	2.95	0.228815	
Ctrl	F1	80.88	2.73E-18	***
Ctrl	F2	15.88	0.000355	**
Ctrl	F3	80.63	3.10E-18	***
Ctrl	F4	29.30	4.33E-07	***
Ctrl	F5	48.69	2.67E-11	***
Ctrl	F6	29.82	3.35E-07	***
Ctrl	NOVOS_NCS	464.11	1.70E-101	***
Ctrl	F1_NCS	435.06	3.37E-95	***
Ctrl	F2_NCS	231.94	4.31E-51	***
Ctrl	F3_NCS	372.04	1.63E-81	***
Ctrl	F4_NCS	307.72	1.51E-67	***
Ctrl	F5_NCS	262.90	8.16E-58	***
Ctrl	F6_NCS	367.62	1.48E-80	***
NOVOS	NOVOS_NCS	404.90	1.19E-88	***
F1	F1_NCS	76.94	1.96E-17	***
F2	F2_NCS	185.72	4.69E-41	***
F3	F3_NCS	152.81	6.57E-34	***
F4	F4_NCS	228.08	2.98E-50	***
F5	F5_NCS	118.35	2.00E-26	***
F6	F6_NCS	225.01	1.38E-49	***
Ctrl_NCS	NOVOS_NCS	129.18	8.90E-29	***
Ctrl_NCS	F1_NCS	782.41	1.3E-170	***
Ctrl_NCS	F2_NCS	290.19	9.66E-64	***
Ctrl_NCS	F3_NCS	199.47	4.84E-44	***
Ctrl_NCS	F4_NCS	253.88	7.44E-56	***

Ctrl_NCS	F5_NCS	255.25	3.73E-56	***
Ctrl_NCS	F6_NCS	184.32	9.44E-41	***

**: p-value < 0.0003; ***: p-value < 3E-05 - These two values correspond to the Bonferroni-corrected significance levels 0.01, 0.001.

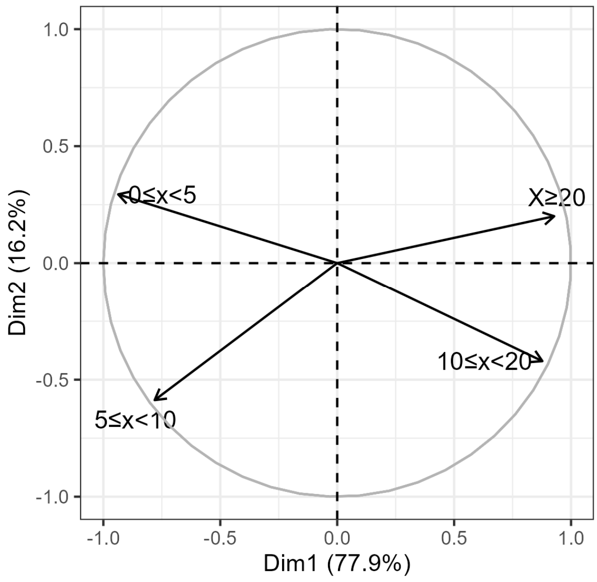

Figure S1. Loading plot showing the correlation between each of the original variables describing the foci distribution and the first two components resulting from the PCA. More cells with 10 or more foci result in a higher value of PC1, while more cells with less than 5 or more than 19 foci result in a higher value of PC2.

Table S3. Number of ingredients in each formulation and corresponding weighted Euclidean distance from the centroid of the "non-damaged cells" cluster. A lower distance suggests a higher effectiveness of the formulation.

**ID**	**Ingredients number**	**Euclidean distance**
Ctrl	-	5.01
NOVOS	12	3.35
F1*	3	2.38
F2*	4	1.99
F3	4	2.48
F4*	3	2.18
F5	5	2.14
F6	5	2.73

* Formulation selected for further testing
